# Engaging the Arts for Wellbeing in the United States of America: A Scoping Review

**DOI:** 10.3389/fpsyg.2021.791773

**Published:** 2022-02-09

**Authors:** Virginia Pesata, Aaron Colverson, Jill Sonke, Jane Morgan-Daniel, Nancy Schaefer, Kelley Sams, Flor Maria-Enid Carrion, Sarah Hanson

**Affiliations:** ^1^University of Florida Center for Arts in Medicine Interdisciplinary Research Lab, University of Florida, Center of Arts in Medicine, Gainesville, FL, United States; ^2^Health Science Center Libraries, University of Florida, Gainesville, FL, United States; ^3^School of Public Health and Tropical Medicine, Tulane University, New Orleans, LA, United States; ^4^UF Department of Surgery, University of Florida, Gainesville, FL, United States

**Keywords:** arts in health, wellbeing, scoping review, evidence synthesis, health outcomes, marginalized populations, public health

## Abstract

There is increasing interest today in how the arts contribute to individual and community wellbeing. This scoping review identified and examined ways in which the arts have been used to address wellbeing in communities in the United States. The review examined 44 publications, with combined study populations representing a total of 5,080 research participants, including marginalized populations. It identified the types of artistic practices and interventions being conducted, research methods, and outcomes measured. It highlights positive associations found across a broad spectrum of psychological, physical, and social outcomes, including improvements in self-esteem and identity formation, cognition, physical balance, and physical conditioning. It also reports negative outcomes of arts interventions that may be underreported. The study identifies the need for core outcomes sets and reporting guidelines for advancing evidence synthesis in this area.

## Introduction

There is increasing interest across the health sciences in how the arts contribute to health and wellbeing, as evidenced by initiatives such as the World Health Organization’s Global Arts and Health Program and an upsurge in evidence synthesis, including scoping and systematic reviews (Fancourt and Finn, 2019; Golden et al., 2019; World Health Organization [WHO], 2021). This article reports the results of a scoping review on how the arts have been used in the recent past to address wellbeing in communities in the United States.

Wellbeing and the arts are both difficult to define, and neither concept has a single universally accepted definition. This lack of definition results naturally from the breadth and complexity of each domain, yet presents significant challenges to research—both within and at the intersections of the domains.

The challenge of defining wellbeing and identifying its implications for research have been frequently noted and numerous definitions or frames for wellbeing have been offered (Ryff and Keyes, 1995; Carlisle and Hanlon, 2008; Dodge et al., 2012; Naidoo, 2019). Dodge et al. (2012) reviewed attempts to define the concept in scholarly literature and proposed a definition of wellbeing as “the balance point between an individual’s resource pool and the challenges faced” (p. 230). This definition centers on the fluctuating balance of challenges and resources across psychological, physical, and social dimensions. Other definitions focus on how individuals evaluate their own lives (Diener, 2000). For example, the Robert Wood Johnson Foundation (RWJF) describes wellbeing as “the comprehensive view of how individuals and communities experience and evaluate their lives, including their physical and mental health and having the skills and opportunities to construct meaningful futures” (Robert Wood Johnson Foundation [RWJF], 2019). Similarly, relational wellbeing represents an interchange between personal, social, and environmental processes that is socially and culturally created and occurring in a particular time and place (Atkinson, 2013; White, 2015; Tan, 2020). In the recent World Health Organization (WHO) report on the arts, health and wellbeing, the concept is framed subjectively to include “affective wellbeing (positive emotions in our daily lives), evaluative wellbeing (our life satisfaction) and eudemonic wellbeing (our sense of meaning, control, autonomy, and purpose in our lives)” (Fancourt and Finn, 2019, p. 21).

Similarly, and understandably, “the arts” also defy common definition. The arts have many forms and interpretations; and represent experiences that are also subjectively defined. Fancourt and Finn (2019), however, offer several fundamental and cross-cultural characteristics of the arts, including presence of an object or experience that is valued beyond its utility, imaginative experiences, and emotional involvement. Davies et al. (2015) offer a working definition in the form of a classification of art forms specifically for research on the arts in relation to health, including: performing arts; visual arts, design, and craft; community/cultural festivals, fairs and events; literature; and online, digital and electronic arts. These categories were created specifically for studies of arts in health, and those that seek to explore causal pathways that link the arts to health outcomes. This definition recognizes a range of engagement with the arts, from participatory art making to listening, viewing, or watching. Recognizing that passive is a flawed term in this context-as listening and watching are not fully passive, but invite active cognitive, emotional, and other forms of engagement and participation–the terms active participation and receptive participation are used to represent this distinction (Davies et al., 2016; Story et al., 2021).

Interest in the arts as a means for promoting health and enhancing wellbeing has expanded significantly in recent decades. A recent World Health Organization report presented a scoping review of over 3,000 publications that investigate the arts in relation to health and wellbeing (Fancourt and Finn, 2019). In the United Kingdom and several other countries, arts activities are prescribed within national or regional social prescribing, or “arts on prescription,” programs (Polley et al., 2017; Chatterjee et al., 2018). Outcomes of these programs include improved wellbeing, physical and mental health, social support, and management of health-related conditions, as well as health care cost reduction and decrease in emergency room use (Chatterjee et al., 2018; Drinkwater et al., 2019; Fancourt and Finn, 2019; Redmond et al., 2019). While several state or regional pilots are currently under way, no national social prescribing structures have been developed in the United States.

There is also a growing body of research exploring how the arts contribute to wellbeing at the community level. Recent studies and reports suggest that arts and cultural practices enhance social cohesion, preserve culturally relevant social capital, and contribute to healthy communities, as well as to individual wellbeing (Landry, 1996; Daykin, 2012; Gillam, 2018; Sonke et al., 2019; Vougioukalou et al., 2019; Engh et al., 2021). The 2017 Survey of Arts Participation in the United States, a nationally representative survey, reported that 54% (*n* = 133 million) of US adults attend creative, arts or cultural activities, 54% create or perform art, 57% read short stories, novels, poems or plays, and 74% use electronic media to consume artistic content (National Endowment for the Arts [NEA], 2019). Analyses of data from the General Social Survey also confirmed a social gradient in arts participation in the United States, with greater disparity in attendance at arts events than in participation in arts activities (Bone et al., 2021a). In a recent statistical study, Bone et al. (2021b) found positive associations between participation in arts groups and elements of evaluative, experienced, and eudaimonic wellbeing in the United States population.

The field of arts in health in the United States has focused strongly on the use of the arts in clinical settings. Many studies and reviews globally include reports on arts interventions that take place in the clinical setting (e.g., hospitals, clinics, and long-term care) such as Deatrich et al. (2016), Cosio and Lin (2018), Fancourt and Finn (2019), Golden et al. (2019), and Ambler et al. (2020). However, work is also being done throughout the country that engages the arts to address wellbeing outside of clinical settings, at the population and community level. For this reason, this review was designed to include only studies that occurred in the community with a focus on wellbeing and in a public health context. From a public health perspective, wellbeing contributes to disease prevention and health promotion by integrating physical and mental health (Dunn, 1973). Understanding of how wellbeing contributes to disease prevention and health promotion builds upon the World Health Organization’s (WHO) definition of health as a “state of complete physical, mental and social wellbeing and not merely the absence of disease and infirmity” (1948, p. 1).

The overall aim of this scoping review was to identify and examine ways in which the arts have been used to address wellbeing in communities in the United States. The objectives were to identify the types of artistic practices, tools used, and interventions being conducted to address wellbeing in communities.

This review describes study populations, research designs, art forms, wellbeing frameworks, interventions, and outcomes. Studies were included that either describe their purpose as addressing wellbeing or were determined by the researchers to address one or more of the key components of wellbeing: psychological, physical, or social. The review also identifies gaps in literature and offers recommendations for future work in the field.

## Methods

The scoping review was guided by the following research question: *how have the arts been used to address wellbeing in communities in the United States*? The review used the Dodge et al. (2012), Fancourt and Finn (2019), and Robert Wood Johnson Foundation [RWJF] (2019) definitions of wellbeing to form a multidimensional, but clear, framing. This framing encompassed wellbeing on the individual level as balancing challenges and opportunities in one’s life via psychological, physical, and social components (Dodge et al., 2012), and also encompassed dimensions of subjective wellbeing, affective wellbeing, evaluative wellbeing, and eudemonic wellbeing (Fancourt and Finn, 2019).

The review also engaged a broad and inclusive concept of the arts, drawing on Davies et al.’s (2012) categorizations to include performing arts; visual arts, design, and craft; community/cultural festivals, fairs and events; literature; and online, digital and electronic arts.

Community setting was defined to include settings such as community centers, parks, arts facilities, schools, prisons, etc., and to exclude programs taking place in settings such as hospitals, health centers, and long-term care facilities. For this review, a community was considered as a group inhabiting a common geographic place, having one or more common ties, or sharing a common identity-forming narrative (Hillery, 1982; Lowe, 2021). Within this definition, it is recognized that communities of people share common geographic areas, including those composed of culturally distinct members and culturally heterogeneous groups. Transient communities are also recognized, including temporary, resettled, dispersed, or displaced residents, and including migrant, diasporic, or student communities.

This scoping review examines literature that specifically states a focus on improving or addressing wellbeing through the arts. It focuses on work undertaken in the United States, excluding the territories and protectorates, with the purpose of capturing evidence from programs that are being conducted under similar social and policy contexts. The search was restricted to publications in the past 7 years to capture the most recent work, and to focus on identification of current challenges, opportunities, and considerations relevant to future work in this area.

### Study Eligibility

The review was guided by The Joanna Briggs Institute’s (JBI) Methodology for JBI Scoping Reviews (Peters et al., 2017). This methodology provides an overview of evidence in a research area, with a systematic process for literature searching, screening, and analysis. It was conducted as one of two scoping reviews that were part of a larger project focusing on the use of the arts in community health settings in the United States. The first scoping review examined arts-based programs in health communication (Sonke et al., 2021b). A protocol for the entire project, inclusive of both reviews, was registered with the JBI in January 2019, under the preliminary title “Engaging the Arts in Health Communication and Wellbeing: A Scoping Review.”

#### Study Inclusion and Exclusion Criteria

The PICOS framework was used to identify inclusion criteria:

Population (P): People of all ages participating in a community setting in the United States. The community setting was defined as outside of a health facility, but it could take place in a senior living community, prison, school, etc.; it could be conducted by a hospital, but in a community setting.

Intervention (I): The intervention was an arts-based intervention, program, or practice intended to address wellbeing.

Comparator (C): Studies comparing arts interventions to other interventions, and studies with no comparator were included.

Outcomes (O): All wellbeing outcomes were included. Those outcomes that assess affective wellbeing, evaluative wellbeing, and eudemonic wellbeing in the individual and community were eligible for inclusion.

Study Design (S): All research designs were included. Reports evaluating programs were included. Systematic and scoping reviews were excluded.

This review focused on work done in the recent past and was limited to studies published on or after 1 January 2013, with interventions concluding after January 2008. Only papers determined by the research team to be from credible sources were considered. These included articles from scientific journals, as well as reports from professional organizations, governmental, and non-governmental organizations, or agencies (i.e., the World Health Organization). Doctoral dissertations and master’s theses were also evaluated against inclusion/exclusion criteria, and only English language full text publications were included.

#### Data Sources

Two health sciences librarians developed and conducted the literature search (JMD and NS). An initial search for existing related systematic and scoping reviews or protocols was carried out on 24 August 2020. The keyword search strategy (art OR arts OR artistic OR artist OR artists) AND (wellbeing OR “wellbeing” OR “wellbeing”) was used in BioMed Central Systematic Reviews, Campbell Collaboration Education Group, Cochrane Database of Systematic Reviews, Cochrane Public Health Review Group, JBI Systematic Review Register, JBI Evidence Synthesis, and PROSPERO: International Prospective Register of Systematic Reviews. Most of the four related registrations from the previous 7 years focused on Australia or the United Kingdom; none focused solely on the United States. In addition, none of the reviews or protocols examined how the arts were being used to address wellbeing at a broad community-level.

#### Search Strategy

The base search strategy for the full literature review was developed iteratively. This search was conducted in seven databases that focused either on the arts or health: PubMed; Web of Science; ProQuest’s Applied Social Sciences Index and Abstracts; and EBSCOHost’s Alt HealthWatch, Art and Architecture Source, CINAHL, and APAPsycINFO. On 31 August 2020, the final literature search exploited subject headings (when such headings were offered in a database) and keywords for each concept mentioned above in the Data Sources section, as well as terms for community (non-healthcare facility) settings and geographic locations within the United States (though not specifically its territories and protectorates.) When a database contained no relevant subject heading for a concept, the search consisted only of keywords for that concept. All keyword searching for a concept involved truncated and phrase-searched terms in title and abstract fields. Where possible, results were limited to the United States (including specific states and some specific cities), publication dates 1 August 2013, onward, and English language full text (PubMed Search Terms, Supplementary Table 5).

The 1,729 results were exported from the databases into EndNote and de-duplicated. In preparation for screening, the remaining 1,278 unique references were exported into Covidence. Subsequently, the project team hand-searched the following web archives and databases: National Organization for Arts in Health (NOAH), Alliance for the Arts in Research Universities (a2ru), American Art Therapy Association, American Music Therapy Association, the University of Florida Center for Arts in Medicine Research Database, and the National Endowment for the Arts. Three hundred and eighty additional references were added to Covidence from the hand-search. After automated de-duplication from within Covidence, the final total number of references for title/abstract screening was 1386.

#### Study Selection

Title and abstract and full text screening were completed by the research team members in independent blinded pairs using Covidence. Agreement between two reviewers was required with a third reviewer resolving discrepancies, as needed.

#### Data Extraction

The authors developed a screening form in Covidence’s data extraction tool. The research team extracted the data (A.C., M.C., and S.H.), and it was checked for accuracy and completeness by a separate author (V.P.).

## Results

### Numerical Summary

After title and abstract and full text screening by AC, JS, VP, MC, and SH, 44 studies were included in the final review. Of these, there were 11 doctoral dissertations, 1 master’s thesis, and 5 reports. The remaining 27 records were original research articles (PRISMA Diagram, Supplementary Figure 1).

### Sample Size and Populations

The combined study populations of the included results represented a total of 5,080 research participants. The sample size ranged from 8 to 1,198 with a mean of 134 and a median of 21. Additionally, 178,000 participants were included in an evaluation report (Pourat et al., 2018). The various target populations were described by race/ethnicity (e.g., Indigenous, Black, African American, Latinx, Chinese, Filipino, and Burmese), age (e.g., older adults, adolescents, youths, students, and adults), profession or activity (e.g., laborers, farmers, musicians, and dancers).

Most articles (*n* = 34) focused on how arts interventions were used to improve wellbeing in marginalized populations. The study used two complementary concepts to form its definition for marginalized populations. The first oriented toward power imbalance, “marginalization as a multidimensional, dynamic, context-dependent, and diverse web of processes, rooted in power imbalance and systematically directed toward specific groups and individuals, with probabilistic implications for development” was used for the analysis (Causadias and Umaña-Taylor, 2018). And the second oriented toward identities, recognizing exclusion due to gender identity, race, age, sexual orientation, physical ability and/or immigration status (Baah et al., 2019). For the analysis, the definitions given by the study authors were used. Older adults were considered those 55–64 years old, and elderly were considered those 65 or older.

Populations identified in the studies included indigenous (*n* = 5), African American/Black (*n* = 7), immigrant (*n* = 5), elderly or older adult (*n* = 7), young adult (*n* = 2), youth and children (*n* = 2) and women and girls (*n* = 7). For example, Ka’Opua et al. (2016) addressed intergenerational connection and cultural resilience between native Hawaiian young adults and elders through an active-participatory storytelling curriculum. Kitwana (2014) used ethnographic methods and active-participatory dance to explore holistic wellbeing (psychological, physical, and social) needs in African American/Black adults (*n* = 7). Mares et al. (2020) addressed self and collective perceptions of felt and real structural forms of violence and vulnerability in Latinx day-farmworkers in Vermont through a combined comic-book and gardening intervention. Harrison et al. (2020) addressed perceptions, beliefs, opinions, and attitudes about physical activity and exercise in 58 urban-residing elderly minority individuals. Brown (2017) used the Social Justice Youth Development framework to address trauma, racialized gender oppression, and healthy development in African American/Black youth through an artistic expression program designed to facilitate healing. Deng (2017) investigated the effects of museum-based experiences on learning and behavioral outcomes in children with autism. Finally, McConnell et al. (2016) addressed gendered oppression through semi-structured interviews with participants of the Michigan Womyn’s Music Festival (Table 1).

**TABLE 1:** Marginalized populations.

Populations	Number	References
Indigenous	5	Anguluan-Coger, 2013; Ka’Opua et al., 2016; Mitchell, 2016; Lamar, 2019; Doğan and Timothy, 2020
Elderly/Older adult	7	Gooding et al., 2014; Moore et al., 2017; Cantu and Fleuriet, 2018; Fleuriet and Chauvin, 2018; Harrison et al., 2020; Johnson et al., 2020; Strong and Midden, 2020
Immigrant	5	Stephenson et al., 2013; Novak, 2016; Fleming et al., 2017; Yam, 2017; Mares et al., 2020
Women and girls	6	Moe, 2014; O’Brien, 2015, 2016; McConnell et al., 2016; Travis et al., 2016; Teti et al., 2017
Young adult	2	Travis and Bowman, 2015; Porias, 2020
African American/Black	7	Kitwana, 2014; Brown, 2017; Camp, 2017; Schroeder et al., 2017; Atkins et al., 2018; Campbell, 2019; Addie et al., 2020
Children/Youth	2	Ja, 2014; Deng, 2017
Total	34	

### Study Designs

Study designs were identified by the researchers and coded by VP and AC. Two broad categories emerged: outcomes research studies and descriptive studies.

### Outcomes Research

The first study design category, outcomes research, included studies that measured results of an arts intervention or reported results of being engaged in the arts on wellbeing. This category included the subcategories of evaluation, community-based participatory research (CBPR), experimental, correlational, and model testing.

### Evaluation Studies

The seven evaluation studies appraised programs or curricula. Outcome evaluation studies are designed to measure the program goal. These designs can involve both quantitative and qualitative methods. All these studies reported psychosocial and cohesion outcomes. For example, Brown (2017) evaluated a “Healing Space of Refuge” youth development program for Black youth and found that the program contributed to cultural cohesion and improved family relationships. Ka’Opua et al. (2016) evaluated an oral history curriculum for school-aged children utilizing native Hawaiian elders’ stories, finding increased knowledge of social responsibility and intergenerational connection.

### Community-Based Participatory Research

Nine studies used community-based participatory research methods as identified by the studies’ authors. This research approach is often used by public health organizations with populations that experience marginalization. The community-based participatory research (CBPR) approach involves a process wherein researchers and community stakeholders act as equal partners in the research process. The goals generally are educating the community, improving practice, or social change (Tremblay et al., 2018). Of these methods, participatory action research, community-based participatory research, or Photovoice were identified by the study authors. The CBPR articles concentrated on community involvement and assessment for the development of programs (*n* = 2), evaluation of art-based programs (*n* = 3), and to understand the health and wellbeing of a population (*n* = 4). Of note, MacAulay et al. (2019) utilized CBPR to develop and evaluate a music training program for older adults, finding statistically significant (*p* < 0.05) improvement in executive function, global cognition, verbal fluency, visual memory performance, self-efficacy, and emotional wellbeing.

There were four studies that used Photovoice methodology. Photovoice is “a public health strategy in which underserved individuals use photography and narratives to identify, record, and share their personal and community health experiences” (Catalani and Minkler, 2010). Teti et al. (2017) used Photovoice to provide mental health support for women living with HIV. This was found to facilitate expressing emotions, address distress and process trauma, which led to empowerment.

### Experimental Studies

Experimental studies are used to establish evidence of causation. Essential elements include manipulation of the independent variable, a control group, and random assignment of subjects to a study group (Gray and Grove, 2021). The three randomized control trials included two studies of older adults using, drama, and choir interventions (Moore et al., 2017; Johnson et al., 2020). These interventions produced findings of increased self-esteem, confidence, happiness and interest in life, and decreased loneliness. A study of a 4-week journaling intervention of parents found a decrease in negative affect (Ahmed, 2017).

### Correlational and Model Testing Studies

Correlational studies establish the strength and direction of relationships between variables (Gray and Grove, 2021). There were four correlational studies. A longitudinal correlational study on musical training on cognition in older adults was conducted by Gooding et al. (2014). A correlation was found between the ability to read music and increased performance on the Animal Naming Test (ANT; semantic verbal fluency) and Logical Memory Story A Immediate Recall (LMI; episodic memory) measures. An association was found between cognitive reserve and improved late-life episodic and semantic memory.

Additionally, Strong and Midden (2020) compared cognition of adult instrumental musicians (active vs. former) and non-musicians. A standardized neuropsychological battery and self-reported measures of levels of physical activity, social activity, and overall health were used. All musicians (active and former) had higher scores on the Boston Naming Test and Controlled Oral Word Association compared to non-musicians. Active musicians scored highest on the Stroop task (Delis-Kaplan Executive Function System: Color Word Interference), indicating that cognitive benefits (in the domain of language) of early life music lessons continue into late life.

Model testing is correlational research methods that measures proposed relationships in a theoretical model (Gray and Grove, 2021). A study by Meeks et al. (2018) tested flow state as a model of psychological benefit from theater, finding that theater had an indirect effect on individual and community wellbeing across all ages. Travis and Bowman (2015) used a comparative correlational design to test a measure of empowerment and risk in association with music engagement and its application to the individual and community.

### Descriptive Studies

Descriptive studies describe the characteristics of an individual, situation, group, or population (Gray and Grove, 2021). A total of 17 descriptive studies focused on describing program participants’ perceptions, motivations, meaning, and attitudes in relation to arts interventions. These studies had quantitative (*n* = 3), qualitative (*n* = 5), and mixed methods (*n* = 6) designs and included ethnographies (*n* = 3) and reports (*n* = 5, the reports are not counted as studies). For example, Ja’s (2014) study of high school students found that students participating in performing and fine arts programs reported higher autonomous motivation than students in academic clubs. Doğan and Timothy (2020) evaluated the effect of ecomuseums on the Ak-Chin tribal community, finding positive social and cultural benefits for the entire community.

Ethnographies (*n* = 3) included a study of West African dance on mental health that found improvements in emotional regulation, maintaining presence, and addressing diasporic stressors (Kitwana, 2014). McConnell et al. (2016) explored the Michigan Womyn’s Music Festival and found it promoted adaptive responding, including safety, empowerment, self-expression, and sense of community with other women.

### Reports

The reports in this review identified projects and studies which met the protocol criteria. Five reports were not research studies but did evaluate programs (O’Brien, 2016; Pourat et al., 2018; Campbell, 2019; Lamar, 2019; Sonke et al., 2019). Programs included Healing in Motion which used Dance Movement Therapy to help heal Post Traumatic Slave Syndrome (Campbell, 2019). Similarly, the Move 2 Love dance program was developed to support physical and social wellbeing in older women and has operated for over 23 years. The evaluation reported addiction avoidance, increased physical activity, and increased social and psychological wellbeing (O’Brien, 2016).

Lamar (2019) found that a program to connect museum art collections to the native peoples whose culture the art collection represented increased community wellbeing. Pourat et al. (2018) evaluated Parks After Dark, a program designed to increase access to high quality recreational activities in poorer neighborhoods of Los Angeles County. The authors reported positive changes in all six of their goals, including lowered rates of violence and greater perceptions of safety among participants, as well as increased physical activity, social cohesion, and perceptions of community wellbeing.

A report by Sonke et al. (2019) presented art programs in the United States that addressed collective trauma, racism, mental health, chronic disease, social exclusion, and social isolation. The programs described cross sector collaboration between community development, public health, and arts and culture (Table 2).

**TABLE 2:** Study designs.

Study design	N	Levels of evidence (JBI)
**Outcomes research**		
Evaluation	7	Level 3.e
Community-based Participatory research (CBPR) evaluative	3	Level 3.e
RCT	3	Level 1.c
Correlational	4	Level 3.e
**Descriptive studies**		
Qualitative, quantitative, and mixed methods	14	Level 4.c, 4.d
CBPR-photovoice-descriptive	3	Level 4
CBPR-descriptive-other methods	2	Level 4
Ethnographies	3	Level 4
Reports	5	Level 5
**Total**	44	

### Art Forms and Types of Participation

The arts forms used in the interventions were grouped either by a specific type of art form (e.g., music, dance, theater, and literary) or mixed art forms (i.e., two or more art forms). Additionally, they were grouped by type of involvement, either active participation or receptive participation (Turino, 2008; Story et al., 2021).

### Active Participation

Active participation was the most prevalent mode of arts involvement in the reviewed studies (*n* = 38). Active participation by dancing, playing an instrument, singing (music), acting in a theater production, or journaling (literary art) are examples. Art forms included mixed arts (*n* = 14), dance (*n* = 9), music (*n* = 8), theater (*n* = 3), literary arts (*n* = 2), and storytelling (*n* = 2). Participation in dance programs (*n* = 9) were found to be a low cost, enjoyable and culturally appropriate means to accessible physical activity for children and adults with positive benefits to physical, social, and psychological wellbeing (Kitwana, 2014; Moe, 2014; Feinberg et al., 2016; O’Brien, 2016; Schroeder et al., 2017; Atkins et al., 2018; Campbell, 2019).

Literary arts interventions included journaling for parents and creative writing for a junior college geology course (Ahmed, 2017; Porias, 2020). Storytelling included oral histories with indigenous elders (Ka’Opua et al., 2016). The mixed active participation art forms included photography/visual arts and storytelling (*n* = 4), weaving and storytelling (*n* = 1), architecture, design, and planning (*n* = 1), multiple arts and cultural activities *n* = 1), music and storytelling (*n* = 1), music and architecture (*n* = 1), music concerts and musicals (*n* = 1), and visual and literary (*n* = 1).

### Receptive Participation

Receptive participation includes watching a performance or engaging in cultural activities (*n* = 6) (Small, 1998; Turino, 2008). Of the art forms with receptive participation, cultural activities i.e., museum visitation (*n* = 3), theater (*n* = 2), and music concerts (1) were included. Overall, participation in these art forms indirectly related to satisfaction and enjoyment of theater events, hedonic wellbeing, social functioning via flow states, sense of belonging, and social engagement. The mixed receptive participation art forms included musical theater and concerts (*n* = 1).

### Both Active and Receptive Participation

This category included studies of activities that engaged people in both active art-making and observation of arts programming. Four studies containing both active and receptive mixed arts participation (e.g., physically dancing and watching a dance performance) were included. Zitcer et al. (2016) gathered qualitative and quantitative data in three adjacent West Philadelphia neighborhoods to determine the strategic value of arts/culture engagement upon community empowerment. In interviews study participants stated the importance of the arts, its value, and the contribution to improve their overall quality of life. Specifically, participants discussed how the arts improve the lives of children. They found that the arts engage children and provide outlets for youth and perceive that this improves the safety of their neighborhoods (Table 3).

**TABLE 3:** Arts forms and participation types.

	N
**Arts forms**	
Mixed	13
Dance	9
Music	8
Theater	5
Storytelling	4
Participation in museums	3
Literary	2
**Total**	44
**Participation type**	
Active	34
Receptive	6
Both	4
**Total**	44

### Units of Analysis

This review considered wellbeing as reported in the included studies at the individual and community levels. In keeping with Dodge et al. (2012), individual wellbeing was framed around three components—psychological, social, and physical. Individual wellbeing that included all three characteristics was considered “holistic wellbeing.” In keeping with Robert Wood Johnson Foundation [RWJF] (2019), community wellbeing was defined as how “communities experience and evaluate their lives.” Each article was coded and categorized as individual or community. Further, the studies were coded as psychological, social, physical, and holistic.

For example, the research presented by Strong and Midden (2020) assessed individual differences between older adult musicians and non-musicians that continue or discontinue playing into late life. The study by Evans and Liu (2019) addressed individual and psychological wellbeing. Their quantitative research investigated the effects of participation in a high school orchestra on members’ self-esteem. In contrast, at the community level, the study by Mares et al. (2020) assessed several arts interventions designed to address structural violence and structural vulnerability that effect the wellbeing of farmworker communities. Of note, participatory dance programs were found to benefit participants on a holistic level. Findings show positive results on physical, social, and psychological wellbeing (Kitwana, 2014; Moe, 2014; Feinberg et al., 2016; O’Brien, 2016; Schroeder et al., 2017; Atkins et al., 2018; Campbell, 2019).

### Outcomes Measured

The included articles reported several types of research outcomes. These outcomes were coded utilizing the taxonomy by Dodd et al. (2018). This taxonomy was used in a recent scoping review (Golden et al., 2019). In this taxonomy the outcomes are divided into a core area and a subdomain. wellbeing outcomes were found in two core areas: Life Impact and Resource Use. Most studies reported outcomes in the Life Impact domain with Emotional Functioning/Wellbeing (*n* = 21) and Social Functioning (*n* = 19) as the most common areas of focus. In the subsequent paragraphs, Life Impact including social, emotional, and physical functioning, global quality of life, and cognitive functioning is followed by Resource Use including economic and hospital/healthcare.

### Life Impact-Emotional Functioning

Emotional functioning is defined as the “impact of disease/condition on emotions or overall wellbeing (e.g., ability to cope, worry, frustration, confidence, perceptions regarding body image and appearance, psychological status, stigma, life satisfaction, meaning and purpose, positive affect, self-esteem, self-perception, and self-efficacy)” (Dodd et al., 2018). Reported outcomes in this area included increases in self-awareness, self-esteem, happiness, confidence, self-efficacy, grit, vitality, sense of self-achievement, experience of flow, positive attitude toward aging, autonomy, competence, motivation, empowerment, psychological safety, and feeling valued, as well as reductions in negative affect and mental health distress (n-21). For example, in a study of the effects of a 6-week Drama Workshop program, Moore et al. (2017) found that participants reported positive changes in happiness, confidence, and self-esteem post-treatment.

### Life Impact-Social Functioning

Social functioning refers to “the impact of disease/condition on social functions (e.g., ability to socialize, behavior in society, communication, companionship, psychosocial development, aggression, recidivism, participation)” (Dodd et al., 2018). The findings in these studies included increased social cohesion, social activity, social connectedness or a decrease in social isolation/loneliness, or social exclusion after attendance in a class or arts intervention (*n* = 19). For example, social interaction skills in children with autism were found to improve after several visits to an art museum (Deng, 2017).

### Life Impact-Physical Functioning

Seven publications reported that arts interventions impacted physical functioning, defined as “physical activities of daily living (for example, ability to walk, independence, self-care, performance status, disability index, motor skills, sexual dysfunction, health behavior, and management)” (Dodd et al., 2018). Participatory dance affected physical functioning in all studies. Outcomes reported included improved physiologic wellbeing, lowered disease burden, prolonged life, more energy, a stronger body, and increasing physical, social, and psychological fitness (Kitwana, 2014; Moe, 2014; Feinberg et al., 2016; O’Brien, 2016; Schroeder et al., 2017; Atkins et al., 2018; Campbell, 2019). For example, Schroeder et al. (2017) studied an intergenerational program for children and adults in an urban community (*n* = 521), finding positive physical benefits as well as increased community engagement and enjoyment.

### Life Impact-Global Quality of Life

Five studies measured global quality of life. In these studies, the measurement was accomplished by using several quality of life and wellbeing measures, such as the Quality of Life AD (Kahle-Wrobleski et al., 2016), Satisfaction with Life Scale (SWLS) (Diener et al., 1985), and the Psychological Wellbeing Scale (PWB) (Diener and Biswas-Diener, 2009). Three of these studies did not find a statistically significant change in global quality of life after the arts intervention. Two studies, Ja (2014) and Meeks et al. (2018), reported a positive increase in global quality of life measures. In a study of theater involvement in Vandenbroucke and Meeks (2018) found a significant difference in global quality of life related to theater involvement (philanthropy and volunteers) and attendance (subscribers and attendances) (All measurement instruments used in studies in this review can be found in Supplementary Table 6).

### Life Impact-Cognitive Functioning

Five studies addressed cognitive functioning, defined as the “impact of disease/condition on cognitive function (e.g., memory lapse, lack of concentration, and attention), outcomes relating to knowledge, attitudes and beliefs (e.g., learning and applying knowledge, spiritual beliefs, and health beliefs/knowledge)” (Dodd et al., 2018). Results reported by Gooding et al. (2014); MacAulay et al. (2019), and Strong and Midden (2020) demonstrated cognitive benefits of music. Cantu and Fleuriet (2018) found that painting, drawing, mixed media, and creative writing enhanced the ability to focus among older adults.

### Resource Use-Economic

This category included articles that assessed utilization of economic resources and hospital use. Two studies assessed resource use relating to food and water. Mares et al. (2020) used storytelling along with community gardening, and a participatory comics and storytelling intervention to address issues of food insecurity and barriers in access to fresh and culturally familiar produce with goals of fostering mental health, participation in a free clinic, and education of the public about farmworkers vulnerability. Mitchell (2016) utilized Photovoice (visual arts and storytelling) to engage members of the Kickapoo Tribe in identifying the effects of water insecurity in their community.

### Resource Use-Hospital/Healthcare

Johnson et al. (2020) measured hospital resource use and other healthcare costs, including hospital use, in their study of a community choir for older adults. They reported a near tripling of health care costs for the control group and a doubling of costs for the intervention group over a 6-month period (Table 4).

**TABLE 4:** Outcomes measured using taxonomy by Dodd et al. (2018).

Domain	Definition	Number
**Life impact**		
Emotional functioning	“The impact of disease condition on emotions or overall wellbeing (e.g., ability to cope, worry, frustration, confidence, perceptions regarding body image and appearance, psychological status, stigma, life satisfaction, meaning and purpose, positive affect, self-esteem, self-perception, and self-efficacy)”	21
Social functioning	“The impact of disease/condition on social functions (e.g., ability to socialize, behavior in society, communication, companionship, psychosocial development, aggression, recidivism, participation)”	19
Physical functioning	“Physical activities of daily living (for example, ability to walk, independence, self-care, performance status, disability index, motor skills, sexual dysfunction, health behavior and management)”	7
Global quality of life	“Includes only implicit composite outcomes measuring global quality of life”	6
Cognitive functioning	“The impact of disease/condition on cognitive function (e.g., memory lapse, lack of concentration, attention), outcomes relating to knowledge, attitudes and beliefs (e.g., learning and applying knowledge, spiritual beliefs, health beliefs/knowledge)”	5
**Resource use**		
Economic	“General outcomes (e.g., cost, resource use)”	2
Hospital/Healthcare	“Hospital: outcomes relating to hospital care”	1

### Negative Outcomes

This review found evidence that arts interventions can have negative outcomes. Moore et al. (2017) found lower anxiety in a control group than those in a drama workshop intervention group. In a study of the effect of listening to rap music on college students, among other findings, Travis and Bowman (2015) reported a higher likelihood of thinking about engaging in sexual activity, drinking alcohol, and marijuana use when listening to rap music than when listening to most other types of music. Surprisingly, there were no completely null findings in any of the publications included in this review (see Extracted Data for All Publications, Supplementary Table 7).

## Discussion

This scoping review was designed using JBI Methodology for Scoping Reviews (Peters et al., 2020). This review type was chosen for the purpose of mapping the key concepts and to “clarify working definitions and/or conceptual boundaries of a topic” (The Joanna Briggs Institute [JBI], 2015). The complexity of the working definitions of both wellbeing and the arts made this task difficult. While there is great interest at present in wellbeing at both the individual and community levels, the lack of universal definitions for the concepts of both wellbeing and the arts poses a significant challenge to evidence synthesis. Given that neither of these concepts is likely to be more clearly defined, the development of Core Outcomes Sets (COS), reporting guidelines, as well as common taxonomies and search strategies for arts in wellbeing research are necessary to increase the possibility for systematic reviews and meta-analyses that can synthesize the increasingly ample evidence and offer insights that can guide investments in the arts for promoting health and wellbeing in communities. These needs have also been noted by other authors (White, 2006; Fancourt and Finn, 2019; Golden et al., 2019; Sonke et al., 2021a). Core outcomes should include healthcare utilization and access, as well as community and individual outcomes.

Outcome measures and subsequent core outcome sets, “aim to improve the consistency and quality of research by providing agreed-upon recommendations regarding what outcomes should be measured as a minimum for a population and setting” (Ramsey et al., 2021). These COS are developed by consensus methods of key stakeholders (Williamson et al., 2012). While COS have been used for many years to guide research and clinical practice and in other disciplines, such and medicine and public health. The use of core outcomes for arts interventions have only recently been considered in relation to public health, and work on core outcomes for arts interventions is currently in the early stages (Smith et al., 2021). The outcomes domains highlighted in this study suggest that arts interventions could be classified in alignment with current outcomes identified in medical taxonomy (Dodd et al., 2018).

As this review suggests, active participation in the arts, particularly in dance and music, has the potential to affect outcomes related to the Dodd et al. (2018) domains of life impact, including social, emotional, and physical functioning, and cognitive functioning. Arts interventions that occur in a group setting may impact social functioning, social isolation, and social cohesion. Impacts of the arts on resource use and hospitalization/healthcare utilization could be further explored.

In keeping with the JBI Methodology, the JBI Levels of Evidence (2014) (The Joanna Briggs Institute Levels of Evidence and Grades of Recommendation Working Party, 2014) were used in this study. Reasonably, recent publications question the use of levels of evidence in relation to arts interventions, due to reliance on quantitative methods (Clift et al., 2021). Use of these levels in this study was not intended to attribute hierarchical value to the studies, their methods, or their findings, but rather to provide an overview of the types of studies being conducted. This study recognizes that qualitative methods are critical to understanding the impact of arts practices and interventions. The JBI Levels of Evidence acknowledges this importance and has implemented the Levels of Evidence for Meaningfulness which includes qualitative and mixed methods designs.

The levels of evidence were also used to assess the overall state of the literature. Because research often advances from descriptive to experimental, the levels of evidence can be used to assess overall research progress in a review (Gray et al., 2017). The majority of the studies in this review were descriptive studies (level 4), but there is an indication of progress toward increasing strength with three studies at level 1 (RCT). This indicates that research on the arts and wellbeing is weak but may be advancing in the United States. However, while the levels of evidence are widely utilized in clinical practice, they may have limited use in other fields. For example, there is no level for public health research such as comparative effectiveness research and studies with designs using large population-based data. Also, there are no clinical indicators for wellbeing in the community setting, which further limits the usefulness of levels of evidence.

This review reports mostly positive associations between engagement in the arts and wellbeing at both the individual and community levels in the United States. The reviewed studies collectively suggest positive associations at the individual level across a broad spectrum of psychological, physical, and social outcomes, with multiple studies highlighting associations between engagement in the arts and improvements in self-esteem and identity formation, cognition, physical balance, and physical conditioning. These effects on wellbeing were more evident with active participation than with receptive participation. Notably, there were more studies involving music and dance than other art forms. These interventions often include group interactions, effecting social wellbeing. This finding is consistent with other reviews (Fancourt and Finn, 2019; Dingle et al., 2021).

There are few other reviews focusing on the arts at the community level. Findings of original research at the community level include improved connection between individuals, often highlighting the significance of intergenerationally and socio-culturally relevant traditions to facilitate these connections. The benefits and value of access to the arts demonstrated in these studies support the need to advance inclusion of arts and cultural activities in education and community development, particularly to improve quality of life and overall wellbeing.

Negative outcomes from arts interventions were found in two studies included in this review. More negative outcomes may occur but may not be found in the literature due to publication bias toward positive outcomes. Gray literature was used in this search in an attempt to balance this bias, but this area requires more consideration in future research.

Social capital and cohesion are increasingly being explored as significant determinants of wellbeing for communities and for individuals within communities, and the arts have been investigated as a means for enhancing social cohesion (Matarasso, 1997; Macnaughton et al., 2005; Ehsan et al., 2019; Sonke et al., 2019; Engh et al., 2021). The findings of this review support the idea that both the availability of arts and cultural resources in communities and targeted arts interventions may be important for promoting individual and collective wellbeing. These results also support the movement toward social prescribing, wherein arts, cultural and social activities can be prescribed by care providers (and sometimes paid for by health systems). Social prescribing programs have been associated with positive health and wellbeing outcomes, and with reducing the burdens on primary and secondary health care systems (Polley and Pilkington, 2017; Drinkwater et al., 2019). Given the different social, economic, political, and healthcare contexts in the nations where social prescribing has been implemented (e.g., the United Kingdom), further research is needed regarding applicability in the United States.

Many of the publications included in this review focus on minority and marginalized populations. While social gradients in arts participation in the United States have been established (Fancourt et al., 2021), indicators of wellbeing among minority and marginalized groups have not been well understood. Previous studies by Ryff (1989, 2017) and Ryff et al. (2003) suggest that minority status may be a positive predictor of eudemonic wellbeing (i.e., purpose in life, personal growth, autonomy, environmental mastery, self-acceptance, and positive relations with others), and that minority-status may uniquely focus and utilize adversities as means to growth-minded behaviors such as finding purpose in life. Ryff (2017) references Keyes (2009), for example, associating higher levels of “flourishing” (low levels of mental distress) among middle-aged Black Americans in contrast to White Americans.

Moreover, collective trauma and racism have been identified as key issues in public health that may well be addressed through arts and culture (Sonke et al., 2019). However, more consideration of minority and marginalized experiences should be brought to studies of the relationships between arts participation and wellbeing, as collective trauma and racism persist in the United States.

This review recognizes several gaps in the literature. Notably, no studies in the review addressed online, digital, or electronic arts. Additionally, the studies included in this review do not represent the entire United States. Two states, California and Pennsylvania were each represented in six studies, and many states were not represented. Reports were included to present arts interventions from more regions.

A lack of standard measures and outcomes was also noted. This may be due to a lack of standardized scales for wellbeing, in general, and certainly points to the lack of core outcomes for arts in public health. Lastly, consistency in reporting is also a challenge to evidence synthesis in this area of focus. Many of the publications did not have significant detail about the art interventions which limits replication of studies and evidence synthesis.

### Limitations

Scoping reviews have limitations. Notably, they do not provide a critical appraisal of publications, which limits generalizability of findings. The aim of a scoping review is to overview the evidence related to a particular question (Munn et al., 2018). While systematic reviews are used in evidence-based healthcare to guide clinical decision making and practice, a scoping review is used primarily to identify types of evidence and examine how research is conducted (Munn et al., 2018). Since there were no other reviews that addressed the use of the arts in wellbeing in the United States specifically, a scoping review of the evidence was deemed an appropriate approach. However, the limits of this approach are recognized in regard to the usefulness of findings.

The review included articles published over a 7-year period and interventions completed within the past 12 years, which limited the volume of findings. As noted, the varied and broad definitions, interpretations and concepts of the arts and wellbeing, posed challenges to the development of search terms and inclusion/exclusion criteria as well as analysis of the evidence. The lack of consistent outcomes measures and variation in research designs was evident. While there are benefits to these varied approaches, they pose challenges to presenting an overview of the evidence. This scoping review was conducted at the beginning of the COVID-19 pandemic, too early to capture work specifically being done around the arts and wellbeing in the pandemic context. There is a growing field of literature on this topic which should be investigated as the pandemic continues to unfold.

## Conclusion

This review suggests that the arts, across a spectrum of forms (e.g., music, dance, and theater) and participatory modalities (e.g., active, receptive, and both), can contribute positively to individual and community wellbeing in the United States. However, heterogeneity in study design limits generalizability between populations, as associations between types of arts participation and wellbeing outcomes differed significantly. Future work including systematic reviews and randomized controlled trials are suggested to narrow gaps in variability between studies and to focus research questions. The study highlights the need for core outcomes sets, and reporting guidelines, and more refined search strategies for advancing evidence synthesis in this area.

## Author Contributions

All authors listed have made a substantial, direct, and intellectual contribution to the work, and approved it for publication.

## Conflict of Interest

The authors declare that the research was conducted in the absence of any commercial or financial relationships that could be construed as a potential conflict of interest.

## Publisher’s Note

All claims expressed in this article are solely those of the authors and do not necessarily represent those of their affiliated organizations, or those of the publisher, the editors and the reviewers. Any product that may be evaluated in this article, or claim that may be made by its manufacturer, is not guaranteed or endorsed by the publisher.
