# Supplementary material for: Engaging the Arts for Wellbeing in the United States of America: A Scoping Review
Source: Front Psychol. 2022 Feb 9;12:791773. doi: 10.3389/fpsyg.2021.791773 (PMC8863598; doi:10.3389/fpsyg.2021.791773)
Supplement: Supplementary file 1 [file Table_1.PDF]

PubMed Search Terms
A base search strategy for the full literature review was developed iteratively. To inform final database selections, preliminary test searching using PubMed and Web of Science Core Collection occurred in August 2020.

Set Number	PubMed Search Terms
1	(wellbeing[tiab] OR "well being"[tiab] OR “well-being”[tiab])
2	(((art[tiab] NOT (antiretroviral[tiab] OR "anti retroviral"[tiab] OR "HAART"[tiab] OR "assisted reproductive therapy"[tiab] OR "assisted reproductive therapies"[tiab] OR "state of the art"[tiab])) OR (arts[tiab] OR artist[tiab] OR artists[tiab] OR artistic[tiab] OR artistry[tiab] OR quilt*[tiab] OR printmak*[tiab] OR "silk screen"[tiab] OR "silk screened"[tiab] OR "silk screening"[tiab] OR acting[tiab] OR actor*[tiab] OR actress*[tiab] OR playwrit*[tiab] OR "playwright*"[tiab] OR jazz[tiab] OR "art-based"[tiab] OR "arts-based"[tiab] OR "creative effort"[tiab] OR "creative efforts"[tiab] OR "creative engagement"[tiab] OR "creative expression"[tiab] OR "creative expressions"[tiab] OR "creative process"[tiab] OR "creative processes"[tiab] OR "creative medicine"[tiab] OR "creative practice"[tiab] OR "creative therapy"[tiab] OR "creative therapies"[tiab] OR "creative writing"[tiab] OR "group writing"[tiab] OR "entertainment education"[tiab] OR "expressive activity"[tiab] OR "expressive activities"[tiab] OR "expressive writing"[tiab] OR "graphic novels"[tiab] OR "journal writing"[tiab] OR "diary writing"[tiab] OR "movement therapy"[tiab] OR "movement therapies"[tiab] OR roleplay*[tiab] OR "role play"[tiab] OR "role playing"[tiab] OR "role-play"[tiab] OR "role-playing"[tiab] OR "role-plays"[tiab] OR animat*[tiab] OR artwork*[tiab] OR ballet[tiab] OR caricature*[tiab] OR cartoon*[tiab] OR choir*[tiab] OR choreography[tiab] OR choreographing[tiab] OR choreographer*[tiab] OR clay[tiab] OR collag*[tiab] OR comic[tiab] OR comics[tiab] OR comicbook*[tiab] OR danc*[tiab] OR drama*[tiab] OR psychodrama*[tiab] OR drawing*[tiab] OR edutainment[tiab] OR film[tiab] OR films[tiab] OR filming[tiab] OR fotonovela*[tiab] OR photonovella*[tiab] OR improvisation*[tiab] OR improvization*[tiab] OR journaling[tiab] OR mandala*[tiab] OR mural*[tiab] OR museum*[tiab] OR music*[tiab] OR novela*[tiab] OR novella*[tiab] OR opera[tiab] OR operas[tiab] OR operatic[tiab] OR paint*[tiab] OR photograph*[tiab] OR photovoice[tiab] OR "photo voice"[tiab] OR pictorial[tiab] OR plays[tiab] OR poet* OR poem*[tiab] OR haiku*[tiab] OR portrait*[tiab] OR

PubMed Search Terms
A base search strategy for the full literature review was developed iteratively. To inform final database selections, preliminary test searching using PubMed and Web of Science Core Collection occurred in August 2020.

	potter*[tiab] OR puppet*[tiab] OR rap[tiab] OR raps[tiab] OR rapping[tiab] OR sculpt*[tiab] OR sing[tiab] OR singing[tiab] OR sings[tiab] OR singer*[tiab] OR skit[tiab] OR skits[tiab] OR song*[tiab] OR story[tiab] OR stories[tiab] OR storyline*[tiab] OR storytell*[tiab] OR textile*[tiab] OR theatr*[tiab] OR theater*[tiab] OR transmedia[tiab] OR watercolor*[tiab] OR "water-color"[tiab] OR "water-colors"[tiab] OR "water color"[tiab] OR "water colors"[tiab] OR watercolor*[tiab] OR ceramics[tiab] OR mosaic*[tiab] OR lyrics[tiab] OR graffiti[tiab] OR "hip hop"[tiab] OR aesthetic*[tiab] OR esthetic*[tiab] OR "spoken word"[tiab] OR sketch*[tiab] OR coloring[tiab] OR video*[tiab] OR movie*[tiab] OR webcast*[tiab] OR "motion picture"[tiab] OR "motion pictures"[tiab] OR cinema*[tiab] OR "electronic game"[tiab] OR "electronic games"[tiab] OR "electronic gaming"[tiab] OR gallery[tiab] OR galleries[tiab] OR "virtual reality"[tiab] OR "virtual realities"[tiab] OR "narrative therapy"[tiab] OR "narrative therapies"[tiab] OR sew[tiab] OR sews[tiab] OR sewing[tiab] OR weave[tiab] OR weaves[tiab] OR weaving[tiab] OR crochet*[tiab] OR knit[tiab] OR knits[tiab] OR knitting[tiab] OR spinning[tiab] OR needlework*[tiab] OR needlepoint*[tiab] OR macram*[tiab] OR embroider*[tiab] OR "rug hooking" OR tapestr*[tiab] OR dyeing[tiab] OR "tie-dy*"[tiab] OR temari[tiab] OR shibori[tiab] OR paperfold*[tiab] OR "paper folding"[tiab] OR origami[tiab] OR scrapbook*[tiab] OR stamping[tiab] OR collage[tiab] OR collaging[tiab] OR collages[tiab] OR decoupage[tiab] OR calligraphy[tiab] OR papercutting[tiab] OR "paper cutting"[tiab] OR papercraft*[tiab] OR paperart*[tiab] OR papercraft*[tiab] OR craft[tiab] OR crafts[tiab] OR crafting[tiab] OR quilling[tiab] OR papermaking[tiab] OR "paper making"[tiab] OR printmaking[tiab] OR marbling[tiab] OR screenprint*[tiab] OR "screen printing"[tiab] OR "paper mache"[tiab] OR "papier mache"[tiab] OR illustration*[tiab] OR whittling[tiab] OR woodcarving[tiab] OR carving[tiab] OR "wood work*"[tiab] OR coopering[tiab] OR cooperage[tiab] OR woodburn*[tiab] OR pyrography[tiab] OR inlay[tiab] OR enameling[tiab] OR cloisonn*[tiab] OR engraving*[tiab] OR embossing*[tiab] OR etching*[tiab] OR "wire work*"[tiab] OR "metal work*"[tiab] OR metalwork*[tiab] OR blacksmith*[tiab] OR smithing[tiab] OR tinsmith*[tiab] OR

PubMed Search Terms
A base search strategy for the full literature review was developed iteratively. To inform final database selections, preliminary test searching using PubMed and Web of Science Core Collection occurred in August 2020.

	goldsmith*[tiab] OR silversmith*[tiab] OR beading[tiab] OR handbuilding[tiab] OR "hand building"[tiab] OR "glass blowing"[tiab] OR glassblow*[tiab] OR "lamp work*"[tiab] OR "stained glass"[tiab] OR basketmak*[tiab] OR basketry[tiab] OR ikebana[tiab] OR "flower arrang*"[tiab] OR "floral arrang*"[tiab] OR "leather work*"[tiab] OR leatherwork*[tiab] OR batik*[tiab] OR lithograph*[tiab] OR "jewelry mak*"[tiab] OR jewelrymak*[tiab] OR stitchery[tiab] OR handicraft*[tiab] OR sandplay*[tiab] OR "sand play*"[tiab] OR sandpaint*[tiab] OR decorat*[tiab]) OR ("Art"[Mesh] OR "Sensory Art Therapies"[Mesh] OR "Art Therapy"[Mesh] OR "Dancing"[Mesh] OR "Gardening"[Mesh] OR "Architecture"[Mesh:NoExp] OR "Interior Design and Furnishings"[Mesh] OR "Photography"[Mesh:NoExp] OR "Printing"[Mesh] OR "Printing, Three-Dimensional"[Mesh] OR "Ceramics"[Mesh:NoExp] OR "Poetry as Topic"[Mesh] OR "Medical Illustration"[Mesh] OR "Video Games"[Mesh] OR "Videotape Recording"[Mesh] OR "Video Recording"[Mesh] OR "Webcasts as Topic"[Mesh] OR "Webcasts"[Publication Type] OR "Instructional Films and Videos"[Publication Type] OR "Motion Pictures"[Mesh] OR "Play and Playthings"[Mesh] OR "Hobbies"[Mesh] OR "Music"[Mesh] OR "Music Therapy"[Mesh] OR "Drama"[Mesh] OR "Psychodrama"[Mesh] OR "Poster"[Publication Type] OR "Television"[Mesh:NoExp] OR "Videodisc Recording"[Mesh] OR "Textiles"[Mesh] OR "Books, Illustrated"[Mesh] OR "Blogging"[Mesh] OR "Color Therapy"[Mesh] OR "Creativity"[Mesh] OR "Fictional Work"[Publication Type] OR "Diary"[Publication Type] OR "Diary as Topic"[Mesh] OR "Graphic Novels as Topic"[Mesh] OR "Graphic Novels"[Publication Type] OR "Portrait"[Publication Type] OR "Portraits as Topic"[Mesh] OR "Drawing"[Publication Type] OR "Narration"[Mesh] OR "Narrative Therapy"[Mesh] OR "Personal Narrative"[Publication Type] OR "Narrative Medicine"[Mesh] OR "Animation"[Publication Type] OR "Caricature"[Publication Type] OR "Pictorial Work"[Publication Type] OR "Caricatures as Topic"[Mesh] OR "Cartoon"[Publication Type] OR "Cartoons as Topic"[Mesh] OR "Architectural Drawing"[Publication Type] OR "Imagination"[Mesh:NoExp] OR "Museums"[Mesh] OR "Paintings"[Mesh] OR

PubMed Search Terms
A base search strategy for the full literature review was developed iteratively. To inform final database selections, preliminary test searching using PubMed and Web of Science Core Collection occurred in August 2020.

	"Poetry"[Publication Type] OR "Radio"[Mesh] OR "Play Therapy"[Mesh] OR "Singing"[Mesh] OR "Imagery (Psychotherapy)"[Mesh] OR "Esthetics"[Mesh] OR "Virtual Reality"[Mesh] OR "Virtual Reality Exposure Therapy"[Mesh] OR "Ceramics"[Mesh] OR "Textiles"[Mesh]))
3	((local[tiab] OR localit*[tiab] OR neighborhood*[tiab] OR neighbourhood*[tiab] OR statewide[tiab] OR communit*[tiab] OR "public health"[tiab] OR "CBPR"[tiab] OR city[tiab] OR cities[tiab] OR municipal*[tiab] OR county[tiab] OR counties[tiab] OR town[tiab] OR towns[tiab] OR village*[tiab] OR suburb*[tiab] OR region*[tiab] OR parish*[tiab] OR diocese*[tiab] OR district*[tiab] OR barrio*[tiab]) OR ("Cities"[Mesh] OR "Suburban Population"[Mesh] OR "Urban Population"[Mesh] OR "Poverty Areas"[Mesh] OR "Local Government"[Mesh] OR "Suburban Health Services"[Mesh] OR "Rural Health Services"[Mesh]))
4	(("united states"[tiab] OR "USA"[tiab] OR appalachia*[tiab] OR "great lakes"[tiab] OR midatlantic[tiab] OR "mid-atlantic"[tiab] OR "mid atlantic"[tiab] OR midwest*[tiab] OR "mid west"[tiab] OR "mid-west"[tiab] OR "mid western"[tiab] OR "mid-western"[tiab] OR Alaska*[tiab] OR inuit*[tiab] OR inupiat*[tiab] OR inupiaq*[tiab] OR aleut*[tiab] OR "na dene"[tiab] OR "na-dene"[tiab] OR eskimo*[tiab] OR Hawaii*[tiab] OR Washington*[tiab] OR Oregon*[tiab] OR California*[tiab] OR Idaho*[tiab] OR Nevada*[tiab] OR Utah*[tiab] OR Arizona*[tiab] OR Montana*[tiab] OR Wyoming*[tiab] OR Colorad*[tiab] OR "new mexico"[tiab] OR "new mexican"[tiab] OR "new mexicans"[tiab] OR Dakota*[tiab] OR Nebraska*[tiab] OR Kansas*[tiab] OR Kansan*[tiab] OR Oklahoma*[tiab] OR Texas*[tiab] OR Texan*[tiab] OR Minnesota*[tiab] OR Iowa*[tiab] OR Missouri*[tiab] OR Arkansa*[tiab] OR Louisiana*[tiab] OR Cajun*[tiab] OR Wiscons*[tiab] OR Illin*[tiab] OR Kentuck*[tiab] OR Tennesse*[tiab] OR Mississippi*[tiab] OR Alabam*[tiab] OR Florida*[tiab] OR Floridian*[tiab] OR Michigan*[tiab] OR Indiana*[tiab] OR Ohio*[tiab] OR Virginia*[tiab] OR Carolina*[tiab] OR Carolinia*[tiab] OR Georgia*[tiab] OR Maine*[tiab] OR Vermont*[tiab] OR Massachusetts*[tiab] OR "New York"[tiab] OR "New Yorker"[tiab] OR "New Yorkers"[tiab] OR "rhode island"[tiab] OR "rhode

PubMed Search Terms
A base search strategy for the full literature review was developed iteratively. To inform final database selections, preliminary test searching using PubMed and Web of Science Core Collection occurred in August 2020.

	islanders"[tiab] OR Connecticut*[tiab] OR "New Jersey"[tiab] OR "New Jerseyans"[tiab] OR Delaware*[tiab] OR Pennsylvania*[tiab] OR Maryland*[tiab] OR "District of Columbia"[tiab] OR "pacific northwest"[tiab] OR "pacific northwestern"[tiab]) OR ("United States" [Mesh]))
5	# 1 AND #2 AND #3 AND #4
6	#5, Filters: English; Publication date from 2013/08/01 onwards
