# Supplementary material for: Engaging the Arts for Wellbeing in the United States of America: A Scoping Review
Source: Front Psychol. 2022 Feb 9;12:791773. doi: 10.3389/fpsyg.2021.791773 (PMC8863598; doi:10.3389/fpsyg.2021.791773)
Supplement: Supplementary file 2 [file Table_2.PDF]

Assessment Instruments

**Measurement categories**	**Instruments**	
Cognitive assessments	Wide-Range Achievement Test-3	Benton Judgment of Line Orientation
	Trail-Making Tests A&B	Animal Naming Test
	Color-Word Interference Trials 1,2,3,4	Logical Memory Story A Immediate Recall
	Weschler Adult Intelligence Scale-IV	NIH Toolbox - Flanker Inhibitory Control and Attention Test
	Digit Span Forward, Backward, and Sequencing trials	Montreal Cognitive Assessment
	Controlled Oral Word Association Test	North American Adult Reading Test
	Boston Naming Test	Uniform Data Set-Neuropsychological Battery 3.0
	California Verbal Learning Test–II	Torrance Tests of Creative Thinking
	Brief Visuospatial Memory Test	Patient Health Questionnaire
	WAIS-III Block Design	
Physical assessments	Community Health Activities Model Program for Seniors	
	California Older Persons Pleasant Events Scale	
	Borg Rating of Perceived Exertion	
	Physical Activity Enjoyment Scales	
	NIH Toolbox - Standing Balance Measure	
Psychological assessments	**Depression/Anxiety scales**	
	Geriatric Depression Scale-Short Form	
	Geriatric Depression Scale	
	Center for Epidemiological Studies Depression Scale-Revised Depression Inventory	
	Beck Anxiety Inventory	
	**Well-being Scales**	

Assessment Instruments

Well-Being Manifestation Measure Scale (WBMMS)<br>Psychological well-being measure (Ryff, 1989)<br>Psychological Well-Being Scale (Diener & Biswas-Diener, 2009)<br>Ryff Scales of Psychological Well-Being
**Empathy/Compassion scales**
Santa Clara Brief Compassion Scale<br>Self-Compassion Scale<br>Toronto Empathy Questionnaire;
**Affect/Mood scales**
Positive and Negative Affect Schedule<br>Bradburn Affect Scale<br>Scale of Positive and Negative Experience
**Quality of life scales**
Quality of Life – AD<br>Satisfaction with Life Scale
**Self-esteem/regulation scales**
Self-Esteem Scale<br>Self-Regulation Questionnaire
**Mixed scales**
NIH Toolbox - Assessment of Neurological and Behavioral Function<br>Parenting Stress Index Short Form, 4th Edition<br>Rap Music Attitudes and Perceptions (RAP) Scale<br>Social Responsiveness Scale<br>Balanced Measure of Psychological Needs<br>NEA Survey of Public Participation in the Arts<br>Harris Burdick Mystery Art Portfolio

Assessment Instruments

Individual and Community
Empowerment Inventory
