# Supplementary material for: Engaging the Arts for Wellbeing in the United States of America: A Scoping Review
Source: Front Psychol. 2022 Feb 9;12:791773. doi: 10.3389/fpsyg.2021.791773 (PMC8863598; doi:10.3389/fpsyg.2021.791773)
Supplement: Supplementary file 3 [file Table_3.PDF]

Extracted Data for all Publications

Reference	Location of study	Purpose of intervention	Art type(s) used	Study population and sample size	Study design	Findings related to well-being	Positive/Negative/Null/Mixed Results	Funding sources
Strong, J. V., & Midden, A. (2020). Cognitive differences between older adult instrumental musicians: Benefits of continuing to play. *Psychology of Music*, *48*(1), 67-83.	Midwest	To determine differences in cognitive ability, physical activity, social activity, and overall health between groups of older instrumental musicians (active and former) and non-musicians.	Music (active participatory)	N=57 (mean age 73.9); former musicians, n=11, five males, mean age 72.6; active musicians, n=32, 21 males, mean age 72.7; non-musicians, n=14, 4 males	Comparative Correlational	No significant group effects found for either episodic memory or visuospatial ability. Active musicians had faster reading speed compared to non-musicians and former musicians; active musicians also showed significantly higher differences in language and executive functioning; former musicians were statistically equivalent.	Mixed (positive and null)	Office of Academic Affiliations, Department of Veterans Affairs.
Kitwana, I. (2014). A Community That Dances Never Dies: An Ethnographic Study on	Illinois	To explore the unique mental health needs of African Americans and expand the understanding of	Dance (active participatory)	N=10 (ages 18-55+); 7 female African Americans from the African dance and drum	Ethnographic	Mental, physical, and spiritual well-being were fostered through culturally relevant	Positive	None given

Extracted Data for all Publications

People of the African Diaspora Within the African Dance and Drum Community in Chicago.		dance/movement therapy concepts outside of the clinical setting.		community of Chicago		community dance. Findings only report on the "majority of participants" and not all.		
Gooding, L. F., Abner, E. L., Jicha, G. A., Kryscio, R. J., & Schmitt, F. A. (2014). Musical training and late-life cognition. *American Journal of Alzheimer's Disease & Other Dementias®, 29*(4), 333-343.	Kentucky	To determine if musical training may be associated with improved late-life episodic and semantic memory as well as a useful marker of cognitive reserve in older adults.	Music (active participatory)	N=237 (age > 60 years); 67.1% female, 7.6% minorities; convenience sample of older adults	Longitudinal Correlational	Higher levels of musical training were associated with improved semantic and episodic memory among highly educated, cognitively normal older adults. Musical training promotes brain plasticity and leads to structural changes with the potential to result in improved cognitive performance across time, even when active participation is no longer present.	Positive	NIA grants P30 AG028383, R01 AG038651, and R01 AG019241

Extracted Data for all Publications

Johnson, J. K., Stewart, A. L., Acree, M., Nápoles, A. M., Flatt, J. D., Max, W. B., & Gregorich, S. E. (2020). A community choir intervention to promote well-being among diverse older adults: Results from the community of voices trial. *The Journals of Gerontology: Series B*, *75*(3), 549-559.	California	To test effects of the Community of Voices choir intervention on the health, well-being, and health care costs of racial/ethnically diverse older adults.	Music (active participatory)	N=390 (mean age 71.3 years) 65% nonwhite older adults	Cluster Randomized Controlled Trial	Involvement in community choirs reduced loneliness and increased interest in life among diverse older adults. No significant group differences were observed for cognitive or physical outcomes or for health care costs.	Mixed (positive and null)	National Institute of Aging at the National Institutes of Health (R01AG042526 and P30AG15272 and the National Center for Advancing Translational Sciences at the National Institutes of Health (UL1 TR000004). Intramural Research Program of the National Institute on Minority Health and Health Disparities.
Schroeder, K., Ratcliffe, S. J., Perez, A., Earley, D., Bowman, C., & Lipman, T. H. (2017). Dance for health: an intergenerational program to increase access	Pennsylvania	To evaluate Dance for Health, an intergenerational program to increase access to physical activity in an underserved, high risk urban community.	Dance (active participatory)	N=521 (ages 2-79 years) 85.8% female, all African American	Evaluation-CBPR	Program had high levels of community engagement and enjoyment. Adequate levels of exertion, particularly for adults, unlike children. No	Mixed (positive and null)	Netter Center for Community Partnerships and the Center for Public Health Initiatives, University of Pennsylvania, The Gamba Family

Extracted Data for all Publications

to physical activity. *Journal of pediatric nursing*, *37*, 29-34.						changes in adiposity (program was low frequency, moderate intensity, and short duration) were reported.		Foundation, Mclean, VA; and the National Institute of Nursing Research (grant number T32NR007100)
Feinberg, J. L., Bowman, C., & Lipman, T. H. (2017). Dance for health: The importance of community engagement and project sustainability. *J. Nurs. Educ. Pract*, *7*, 89-94.	Pennsylvania	To explore the impact of community engagement in Dance for Health on graduate nurse practitioner (NP) students and inner-city high school (HS) students.	Dance (active participatory)	N=44 (ages 8-73 yrs.); 15 nurse practitioner students and 7 high school students, most female; 22 community participants, 70% female	Evaluation-Mixed	Themes: Intergenerational togetherness, Positive environment, increased health awareness, leadership of high school students facilitating dance.	Positive	University of Pennsylvania School of Nursing Office of Nursing Research and the University of Pennsylvania Netter Center for Community Partnerships
Fleuriet, J., & Chauvin, T. (2018). 'Living Other Lives': The impact of senior theatre on older adult well-being. *Journal of Applied Arts & Health*, *9*(1), 37-51.	Texas	To document the impact of participation in the Senior Theater program on older adult well-being	Theatre (active participatory)	N=12 (ages 61-80); 6 male older adults, 3 of Hispanic origin, nine identified as non-Hispanic white	CBPR	Acting contributed to emotional intelligence, self-confidence, self-esteem, social engagement and verbal skills.	Positive	None given

Extracted Data for all Publications

Meeks, S., Shryock, S. K., & Vandenbroucke, R. J. (2018). Theatre involvement and well-being, age differences, and lessons from long-time subscribers. *The Gerontologist*, *58*(2), 278-289.	Kentucky	To determine if participation in theatre (presentational rather than participatory) facilitate flow, positive emotions, and sense of belonging	Theater (receptive participatory)	N = 496 (ages 18-33 = 10.7% and 64+ = 31.5%); 31.5% male, white = 95%; focus groups (N = 20; 6 men; ages 60-77; geographic origin, household income and marital status reflected part 1 demographics)	Model Testing	Theater involvement was indirectly related to satisfaction and enjoyment of the theatre, hedonic well-being, and social functioning, through the psychosocial benefits of flow, social engagement, and belonging. No age differences in the relationship between benefits and well-being.	Mixed (positive and null)	National Endowment for the Arts, Grant #15-3800-7007
Moore, R. C., Straus, E., Dev, S. I., Parish, S. M., Sueko, S., & Eyler, L. T. (2017). Development and pilot randomized control trial of a drama program to enhance well-being among older	California	To test the hypothesis that drama instruction would increase empathy and compassion in late life through a pilot feasibility study	Theatre (active participatory)	N=13 (mean age 77); 71% female, older adults	Randomized Controlled Trial	No pre-to-post-treatment changes in empathy/compassion or mood symptoms were found in either group. Compared to backstage pass group (receptive participation) the drama workshop (active	Mixed (positive and negative)	University of California, San Diego Frontiers of Innovation Scholars Program (FISP) [application number #P3041]; the National Institutes of Health, grant number K23 MH107260),

Extracted Data for all Publications

adults. *The Arts in psychotherapy*, *52*, 1-9.						participation) weekly ratings indicated higher levels of anxiety and lower happiness; however, the drama workshop program had higher self-ratings of positive change in self-esteem, confidence, and happiness post-treatment.		and the Stein Institute for Research on Aging
Addie, Y. O., Strekalova, Y. A. L., & Pufahl, J. (2021). The art and science of systemic wellness in Black communities: Qualitative evaluation of a multimodal theatrical production. *Health Education Journal*, *80*(1), 40-53.	Florida	To evaluate audience reaction and attitudes about historically disenfranchised Black communities in North Central Florida through a play.	Theatre (active participatory)	N=176 (ages 19-91 yrs., M=45.01, SD=19.61); women (N=125, 71%), men (N=47, 27%) and transgenders (N=4, 2%); White (N=85, 48%); Black (N=51, 29%); Hispanic/Latinx (N=12, 6.8%); Asian/Pacific Islander (N=10, 6%); multiracial (N=7, 4%); or	Descriptive	Audience members experienced both immersive moments and low transportation-inducing points that influenced critical reflection on some of the themes addressed in the play. By attending the production, audience members were	N/A	None received

Extracted Data for all Publications

				of Caribbean descent (N=6, 3%). Some selected multiple race categories.		actively participating in civic social wellness and engaged in psychosocial wellbeing.		
Ahmed, S. (2016). *An attitude of gratitude: A randomized controlled pilot study of gratitude journaling among parents of young children* (Doctoral dissertation, Alliant International University).	California	To evaluate the impact of a Positive Psychology Gratitude journaling intervention	Literary arts (active participatory)	N=87 (ages 2-5 yrs.) parents of children were included	Randomized Controlled Trial	Reduced negative affect and overidentification with negative emotions among parents of young children. No significant difference in parenting stress, life satisfaction, depressed mood, and self-compassion.	Mixed (positive and null)	Greater Good Science Center of the University of California, Berkeley
Anguluan-Coger, E. (2015). Intergenerational mythweaving and cultural identity. [Doctoral dissertation, Lesley University]. ProQuest Dissertations Publishing.	Massachusetts	To explore how Indigenous Artivism impacted Filipino-Americans cultural identity and sense of well-being and purpose	Mixed arts (active participatory - visual arts/design & craft, and storytelling)	N=23 (ages 18-86); 12 females, all either first-generation immigrants or naturalized American citizens	Descriptive-CBPR	Greater awareness of cultural identity, well-being and purpose among participants, with mental health implications to other indigenous or immigrant	Positive	None given

Extracted Data for all Publications

						groups, including the promotion of social cohesiveness through the expressive arts.		
Atkins, R., Deatrick, J. A., Bowman, C., Bolick, A., McCurry, I., & Lipman, T. H. (2018). University–Community Partnerships Using a Participatory Action Research Model to Evaluate the Impact of Dance for Health. *Behavioral Sciences*, *8*(12) 113.	Pennsylvania	To evaluate the impact of an urban, intergenerational, and physical activity dance program, and determine priority outcomes with community residents.	Dance (active participatory)	N=25 (ages 43-71); three focus groups: 2 for adults and 1 for youth; 7 adults in first focus group and 12 in second, all female adults and all identified as Black/African American; 6 teens, ages 17-19; 4 youth identified as Black/African American, 2 as Hispanic/Latino, and one as biracial (White and Black/African American); 5 youth were female and one male	Evaluation-CBPR	The most preferred outcome themes were psychosocial and emotional categories of health. For adults, measures of physiologic health associated with managing chronic health conditions were the second most frequently mentioned outcomes. For youth, behavioral outcomes, such as decreased television and computer use and increasing physical activity levels, were the	Mixed (positive and negative)	The Netter Center for Community Partnerships at The University of Pennsylvania School of Nursing and the Gamba Family Foundation, McClean, VA and the Center for Public Health Initiatives, University of Pennsylvania

Extracted Data for all Publications

						second most common outcome.		
Brown, Aishia Ayanna (2016) “Healing Spaces of Refuge”: Social Justice Youth Development, Radical Healing, and Artistic Expression for Black Youth. [Doctoral dissertation, Texas A & M University].	Texas	To examine a youth development program that employed Hip Hop and other artistic expression as a tool to facilitate radical healing in Black youththrough four components (care, critical consciousness, community, and culture).	Mixed arts (active participatory and receptive participatory)	N=41 (ages not given)n=19 Youth Participants n=22 Program staff, teachers, and teaching assistants	Evaluation-Qualitative	Black youth enrolled in the summer arts program provided youth with 1) adults that enact radical care for their personal and community well-being, 2) a space to celebrate their cultural identity, and 3) a culture of critical thinking that assisted them in building their critical consciousness.	Positive	None received
Camp, T. L. (2017). *The Relationship Between Rap Music and the Psychological Well-Being of African American Adolescents*. [Doctoral dissertation, George Mason University].	Virginia	To identify the relationship between rap music and psychological well-being	Music (active participatory)	N=14 (ages not given); Adolescent African Americans	Descriptive-Mixed Methods	Quantitative findings: The study showed that there was no significant relationship between the subscales of rap music and the psychological well-being of African American	Mixed (positive, negative, and null)	None received

Extracted Data for all Publications

ProQuest Dissertations Publishing.						adolescents. There was also no significant difference in the percentage of adolescents by age concerning their attitude and perception of rap music. Qualitative Findings: Themes- rap music provides emotional support, influences behavior; influences attitudes and perception; promotes what appears to be cool and authentic; promotes diverse styles within the rap music genre; and promoting psychological support among rap music. This included both positive and negative behaviors.		

Extracted Data for all Publications

Mitchell, F. M. (2016). Water is life: A community-based participatory study of the significance of water and its relationship to the health and well-being of the Kickapoo Tribe in Kansas. [Doctoral dissertation]. *ProQuest Dissertations & Theses Global.(10240 244).*	Kansas	To engage members of the Kickapoo Tribe in an exploratory investigation of environmental changes in water and its relationship to health and well-being.	Mixed arts (active participatory - visual art and storytelling)	N=11 (ages 35-49); 2 men, all from Kickapoo tribe of Kansas	Descriptive-CBPR-Photovoice	The findings detail how water is fundamental to the lives of the Kickapoo people, and exposes the scope of possible health hazards related to water insecurity in their community.	Positive	Kansas Humanities Council, the CSWE Minority Fellowship Program, the University of Kansas Office of Graduate Studies and the University of Kansas Institute for Social Policy and Research
Moe, A. M. (2014). Healing through movement: the benefits of belly dance for gendered victimization. *Affilia*, *29*(3), 326-339.	Michigan	To document emic (personal) experiences of female belly dancers' views and feelings using belly dance in response to interpersonal harm and/or personal victimization (i.e. intimate partner violence)	Dance (active participatory)	N=67 (ages 22-68); 20 female hobbyist (n=9) and professional (n=11) belly-dancers	Descriptive-Qualitative	Belly dance may be beneficial to empowerment and overall health with a holistic healing component, particularly in terms of gendered interpersonal victimization, self-exploration and discovery.	Positive	(i) Kercher Center for Social Research, Western Michigan University; (ii) Faculty Research and Creative Activities Award, Western Michigan University; (iii) Midwest Sociology

Extracted Data for all Publications

								Society Endowment
Deng, L. (2016). Equity of Access to Cultural Heritage: The Influence of the Museum Experience on Learning in Children with Autism Spectrum Disorder. [Doctoral dissertation, University of South Carolina]. PsychInfo Database. https://scholarcommons.sc.edu/etd/3536	California	To investigate the effects of museum cultural experience on learning and behaviors of children with autism	community and cultural. (Participation in museums) (receptive participatory)	N=10 (ages 8-15); Children diagnosed with Autism Spectrum Disorder	Evaluation-Mixed Methods	Survey-Statistically significant difference between the pre-program and the post-program scores in social communication, social motivation, social responsiveness and social cognition. No difference in social awareness. Observation-increased group communication, making requests for self, task evaluation and answering questions; a decrease in asking questions.	Mixed (Positive, Negative, Null)	None given
Doğan, M., & Timothy, D. J. (2020). Beyond tourism and taxes: the	Arizona	A museum program to aid residents in rediscovering their unique heritage,	Participation in museums (receptive participatory)	N=8 (further demographic data not given)	Descriptive-Qualitative	The ecomuseum is an economic and educational tool to aid	Positive	None given

Extracted Data for all Publications

ecomuseum and social development in the Ak-Chin tribal community. *Journal of Tourism and Cultural Change*, *18*(2), 133-149.		effecting community social wellbeing by preserving elements of the past. Including educating and training the local community.				residents in rediscovering their unique heritage, preserving elements of the past that are on the verge of disappearing, fostering social empowerment and advancing the social wellbeing of the community.		
Evans, P., & Liu, M. Y. (2019). Psychological needs and motivational outcomes in a high school orchestra program. *Journal of Research in Music Education*, *67*(1), 83-105.	Midwest	To investigate the satisfaction and frustration of psychological needs in the context of a high school orchestra program and how they affect music-related and psychosocial outcomes	Music (active participatory)	N=704 (ages not given); high school students in strings programs, 38% boys; further data unavailable	Descriptive-Quantitative	There was a positive prediction of psychological needs satisfaction related to the music program to self-esteem. Self-esteem was negatively predicted by psychological needs frustration related to the program.	Mixed (positive and null)	None received
Ja, N. M. (2014). *School activities and community programs as contexts for*	New York	To assess the effects on well-being by participating in an afterschool activity.	Mixed arts (active participatory and receptive participatory)	N=864 (ages 13-19); 399 males; SES/Race - 378 high SES/White,	Descriptive-Quantitative	Participation in performing and fine arts programs were associated with	Positive	None given

Extracted Data for all Publications

adolescent self-integration, social connection, and well-being: The role of participant characteristics, key features of activity participation, and engagement in reflective practices. [Doctoral dissertation, Cornell University]. ProQuest Dissertations Publishing.				196 high SES/non-white, 121 low SES/white, 142 low SES/non-white		significantly higher autonomous motivation vs. academic clubs and faith-based/community programs. Autonomous motivation was shown to be positively associated with three of six indicators of well-being (life satisfaction, positive affect, psychological resilience).		
Ka'opua, L.S.I., Goodyear-Ka'opua, J.N., Kaawa, J.M., Amona, S.K., Browne, C.V., & Robles, A. S. (2016). Look to the source: Gathering elder stories as segue to youth action-oriented research. *Inter*	Hawai'i	To use storytelling to enhance relational connections between elders and youth, create interest in elder wellbeing, and provide perspectives on diverse social action opportunities	Storytelling (active participatory)	N=21 (6 elders: 4 native Hawaiian, 1 Pacific Islander, 1 European-American; 4 women, 1 male, 1 transgender; 15 youth: 9 males, 9 in the 11th grade, 14 Native Hawaiians)	Evaluation-Mixed Methods	Intergenerational connection is emphasized as fundamental to the development of health-related promotions, particular to indigenous well-being.	Positive	National Institute on Minority Health and Health Disparities (U54MD007584) and the US Administration on Aging, Department of Health and Human Services (90OI0006/01)

national Public Health Journal, *8*(2), 271-282.		for high school students.						
MacAulay, R. K., Edelman, P., Boeve, A., Sprangers, N., & Halpin, A. (2019). Group music training as a multimodal cognitive intervention for older adults. Psychomusicology: Music, Mind, and Brain, 29(4), 180–187. https://doi.org/10.1037/pmu0000239	Maine	To investigate music training in an economical group format in community dwelling older adults, to understand of the relationship between cognitive functioning and psychological well-being with music	Music (active participatory)	N=35 (mean age 70, SD 5.12); older adults, primarily white women (97.6%)	Evaluation-CBPR-Mixed Methods	Improved executive function, global cognition, verbal fluency, and visual memory performance following the intervention. The group provided socialization and camaraderie. Subjective improvements in cognition, self-efficacy and emotional well-being as a result of participating in the music group were found.	Mixed (positive and null)	The University of Maine
Mares, T., Wolcott-MacCausland, N., Doucet, J., Kolovos, A., & Bennett, M. (2020). Using chiles and comics to	Vermont	To impact self and collective perceptions of felt and realized structural violence and vulnerability through	Storytelling (active participatory)	N=20 (further demographic data not given)	Ethnographic	The comics can serve as a vehicle for the transmission of information and the sharing of experiences having to do with health	Positive	City Market Onion River Cooperative, The Vermont Community Foundation, The Harris and Frances Block Foundation, an

Extracted Data for all Publications

address the physical and emotional wellbeing of farmworkers in Vermont's borderlands. *Agriculture and Human Values*, *37*(1), 197-208.		community gardening and comic books				and wellbeing. A series of 20 short-format graphic narratives integrating oral history interviews with migrant workers that focus on the experience of moving to and working in Vermont, including the stresses and mental health impacts of migration and self-care and well-being strategies. This is used with patients and to educate the health care providers and the public.		Anonymous donor via University of Vermont Foundation, and the Ben and Jerry's Foundation. El Viaje Más Caro has received funding from: The Vermont Community Foundation and The University of Vermont Humanities Center
Novak, J. L. (2016). *Considering Cultural Integration in the United States: Empirical Essays on Immigrants' Arts*	United States	To understand the role that arts participation plays in integrating a heterogeneous immigrant population into American society.	Mixed arts (receptive participatory - music concerts and musicals)	N=14 (ages not given); Chinese Americans and Chinese immigrants who live in Chinatown, Chicago; ages not given; sex/gender	Descriptive-Mixed Methods	Results from the National Endowment for the Arts' Survey of Public Participation in the Arts (SPPA)survey: First-generation	N/A	Kip and Mary Ann Hagopian Dissertation Award, the Anne and James Rothenberg Endowed Dissertation Award, scholarship

Participation. [Doctoral dissertation, The Pardee RAND Graduate School]. ProQuest Dissertations Publishing.				identity not given)		immigrants reported lower levels of arts participation than subsequent generations and US born individuals. Cultural integration through expressive and artistic means as a specific dimension of broader civic and social engagement. Participating in arts may offer undocumented immigrants a non-threatening means to engage with their local community and build social capital, fostering means for further social and economic integration.		support from the John and Barbara Vogelstein Foundation, the Agency for Healthcare and Research Quality at the National Institutes of Health (Grant# 1R36HS017531-01 Revised), and the Research: Art Works program at the National Endowment for the Arts (Grant# 14-3800-7012)
O'Brien, E. P. (2015).	Pennsylvania	The purpose of this qualitative study was to	Dance (active participatory)	N=8 (ages 71-81); all female, other	Evaluation-Qualitative	The Fit Dance program was found to	Positive	None given

Extracted Data for all Publications

Positive, active, older but youthful, women, and" FitDance": Uplifting motivation and adherence in community dance exercise. [Doctoral dissertation, Temple University]. ProQuest Dissertations Publishing.		investigate the effect of FitDance, community dance exercise program on elderly women.		demographic data not given		promote vibrant aging, social connections, and well-being by increasing motivation and adherence in community exercise.		
Porias, R. M. (2019). *Creative Writing in Community College Science Students: Mixed Methods Study of Treatment Effects on Active Learning, Creative Thinking, and General Wellbeing with Consideration of Self-Determination. [*Doctoral	California	To ascertain if the creative writing treatments and creativity tasks increased the participants creative thinking capacities and engendered a sense of well-being	Literary arts (active participatory)	N=10 (ages 19-30); multiethnic female and male participants from a junior college intro geology class	Evaluation-Mixed Methods	Findings included pre-post changes in the positive direction for verbal fluency and figural scores on the Torrance Test of Creative Thinking after the creative writing intervention in both group A&B. Creative writing changed the learning atmosphere in the classroom from difficult	Positive	None given

Extracted Data for all Publications

dissertation, Saybrook University]. ProQuest Dissertations Publishing.						for participants to memorize geology-related content to easier to memorize and take exams. Creative writing helped participants recognize their self-worth and confidence, autonomy, and feelings of competence and social relatedness.		
Roygardner, D. (2017). *Letter to the President: Longitudinal Critical Discourse Analysis of Academic and Hip-Hop Genres in a Rap Narrative Program*. [Doctoral dissertation, City University of New York]. ProQuest Dissertations Publishing.	New York	To examine the relationship between the values for Hip hop Narrative and Psychological Well-Being Rap Narratives and expressive and rhetorical strategies.	Mixed arts (active participatory - music and storytelling)	N=12 (ages 13-19); 6 males, majority identified as African American or Latino	Evaluation-Cohort Mixed Methods	Qualitative-Themes included Social-awareness, Self-awareness, Antiphony, Overcoming, Heroism, Prophetic, Relationship skills, Hope, Catharsis, and Literacy. Major findings include that “Hip hop allows for emotional expression.” This theme	Mixed (positive and null)	None given

Extracted Data for all Publications

						emerged as dominant across cohorts and times and associated with relationship skills increasing slightly over time and across cohorts. Quantitative- No significant change scores on the Ryff psychological well-being measure.		
Stephenson, S. M., Smith, Y. J., Gibson, M., & Watson, V. (2013). Traditional weaving as an occupation of Karen refugee women. *Journal of Occupational Science*, *20*(3), 224-235.	Utah	To explore the meaning of weaving for Karen refugee women from Burma as they participated in their traditional practice of weaving	Mixed arts (active participatory) visual arts, design, and craft (weaving) and storytelling	N=8 (ages not given); all Karen refugee women	Descriptive- Qualitative	Weaving contributes the well-being in terms of cultural identity construction, empowerment, social support, and economic survival in a new environment.	Positive	None given
Travis Jr, R., & Bowman, S. W. (2015). Validation of the individual and community	Texas	To assess measurement variance within a new population compared to the original	Music (active participatory)	N=531 (ages 17-25); 57% white, 38% female, all incoming freshmen, and transfers	Correlational	Validation of inventory- strong reliability scores for the individual and community	Mixed (Positive and Negative)	None given

Extracted Data for all Publications

empowerment inventory: A measure of rap music engagement among first-year college students. *Journal of Human Behavior in the Social Environment*, *25*(2), 90-108.		findings of the Travis and Bowman Individual and Community Empowerment Inventory: A Measure of Rap Music Engagement.				empowerment, and empowerment subscales. The subjects identified empowerment and risky behavior-highlighting the potential for Rap music engagement to be simultaneously empowering and risky.		
Travis, R., Bowman, S.W., Childs, J. and Villanueva, R. (2016), Musical Interactions: Girls Who Like and Use Rap Music for Empowerment *Symbolic Interactionist Takes on Music* (*Studies in Symbolic Interaction, Vol. 47*), Emerald Group Publishing Limited, Bingley, pp.	North Carolina	To determine effects of rap music listening habits upon young-female empowerment	Music (active participatory)	N=531 (ages 17-25); all undergraduate students; subsample of female-only undergrads (n = 202; ages 17-18)	Descriptive-Quantitative	Women are more likely to listen to Rap music alone and elicit empowerment from lyrics than in the original study population. Twenty to 30 % answered yes to two areas of risk; thinking about sexual activity and the idea of consuming alcohol.	Mixed (Positive and Negative)	None given

119-149. https://doi.org/10.1108/S0163-239620160000047017								
Vandenbroucke, R., & Meeks, S. (2018). How theatre encourages well-being– and can engage a wider audience. *New Theatre Quarterly*, *34* (4), 374-382.	Kentucky	To determine effects of live theater participation upon health and well-being outcomes in community-dwelling older and younger members	Theatre (receptive participatory)	N=20 (ages not given); all theater attendees, further demographic data not given	Descriptive-Mixed Methods	Regular theater attendance produces psychosocial benefits, contribute to a shared sense of community, connecting individual well-being to community well-being.	N/A	‘Art Works’ grant from the National Endowment for the Arts, #15-3800-7007; Kelly Shryock at the University of Louisville and of Actors Theatre of Louisville, especially Jennifer Bielstein, Steven Knight, Danielle Manley, Erin Meiman, and Justin Williamson
Yam, A. (2017). *Using Photovoice as participatory action research to identify views and perceptions on health and well-being among a group*	Texas	To understand the views and perceptions of health and well-being among a group of resettled Burmese refugees in Houston, Texas.	Mixed arts (active participatory - visual art (photography) and storytelling	N=7 (ages 22-45); 4 females, all Burmese refugees	Descriptive-CBPR-Qualitative-Photovoice	Well-being themes were Family well-being including financial stability, safety/preventive education, ability to understand disease and	N/A	Texas Woman’s University and the School of Health Studies

Extracted Data for all Publications

of Burmese refugees resettled in Houston. [Doctoral dissertation, Texas Woman's University]. ProQuest Dissertations Publishing.						health related material and community engagement. The study identified a need for social systems targeted in these areas.		
Zitcer, A., Hawkins, J., & Vakharia, N. (2016). A capabilities approach to arts and culture? Theorizing community development in West Philadelphia. *P lanning Theory & Practice*, *17*(1) 35-51.	Pennsylvania	To document and analyze the roles of the arts and culture in contributing to health and well-being in West Philadelphia	Mixed arts (active participatory and receptive participatory)	N=45 (ages 25-64); male = 51%, further demographic data not given	Descriptive-Mixed Methods	Most participants saw value in the arts and cited the importance of the arts to improve their overall quality of life (some did not feel this way). Participants also declared they see arts as part of a suite of community building activities.	Mixed (positive and null)	Office of Research, Drexel University, grant number 3776
Harrison, E. A., Lord, L. M., Asongwed, E., Jackson, P., Johnson-Largent, T., Jean Baptiste, A. M., Harris,	Maryland	To understand the perceptions, opinions, beliefs, and attitudes of urban-residing older adults on the benefits of	Dance (active participatory)	N=58 (mean age 72 years); 49 females, 42 African American, 8 Latino, 8 Asian	Descriptive-Mixed Methods	Dance was the preferred physical activity and exercise, fixed or limited income were the main barriers, and	N/A	University of the District of Columbia Agricultural Experiment Station with funding from the Hatch Multistate

Extracted Data for all Publications

B.M., & Jeffery, T. (2020). Perceptions, opinions, beliefs, and attitudes about physical activity and exercise in Urban-Community-residing older adults. *Journal of Primary Care & Community Health, 11*.		physical activity and exercise				prolonged life, more energy, and a stronger body were the main benefits. These interventions are more likely to increase older adults' ability to remain in their communities and improve their overall health and well-being.		Research capacity funding program (NE1439) from the USDA National Institute of Food and Agriculture
Cantu, A. G., & Fleuriet, K. J. (2018). Making the Ordinary More Extraordinary: Exploring Creativity as a Health Promotion Practice Among Older Adults in a Community-Based Professionally Taught Arts Program. *Journal of Holistic Nursing*, *36*(2) 123-133.	Texas	To document psychosocial and mental well-being outcomes of a community-based, arts program for older adults.	Mixed arts (active participatory - visual and literary)	N=138 (ages not given); older adult students, further demographic data not given	Evaluation-Qualitative	Professionally taught arts programming promoted an enhanced ability to focus. Other themes included happiness through the self-confidence generated by creative engagement, improved psychosocial and mental health.	Positive	NIGMS1R01GM088781-01A

Extracted Data for all Publications

Teti, M., French, B., Kabel, A., & Farnan, R. (2017). Portraits of well-being: Photography as a mental health support for women with HIV. *Journal of creativity in mental health*, *12*(1), 48-61.	Missouri	To facilitate group discussion (storytelling) and reflection of living with HIV through photography	Mixed arts (active participatory) visual art (photography) and storytelling	N=30 (ages 18-65); all women, 25 Black/African American; 18 reported income of >$10k/year	Descriptive-CBPR-Photovoice	The project supported women's mental health in four ways by facilitating empowerment and helping women to express themselves, address their mental health with new tools like photography, and process past traumas.	Positive	NIH grants R25 DA28567, R25 HD 045810, R25 MH067127
McConnell, E. A., Todd, N. R., Odahl-Ruan, C., & Shattell, M. (2016). Complicating counterspaces: Intersectionality and the Michigan womyn's music festival. *American journal of community psychology*, *57* (3-4), 473-488.	Illinois	To understand whether and how cultural events and counterspaces facilitate a specific set of processes that promote the well-being of marginalized groups.	Mixed arts (active participatory - music and community and cultural)	N=43 (ages not given); all women, further demographic data not given	Ethnographic	The music festival contributed to participants' experiences of identity and community to promote well-being for members of marginalized groups and liberatory responses to oppression.	Positive	None received
Fleming, P. J., Villa-Torres, L., Taboada, A., Richards,	North Carolina	To understand how the health of male Latino Day laborers	Mixed arts (active participatory -	N=34 (ages not given); all male Latino Day laborers	Descriptive-CBPR-Photovoice	Men's health and well-being were primarily shaped by their	N/A	The Consortium in Latin American and

Extracted Data for all Publications

C., & Barrington, C. (2017). Marginalisation, discrimination and the health of Latino immigrant day labourers in a central North Carolina community. *Health & social care in the community*, *25* (2), 527-537.		was influenced by their experiences in the community and perceptions of their social environment.	visual art and storytelling)			experiences and feelings of discrimination and marginalization.		Caribbean Studies at the University of North Carolina at Chapel Hill and Duke University, among several others
Campbell, B. (2019). Past, present, future: A program development project exploring post traumatic slave syndrome (PTSS) using experiential education and dance/movement therapy informed approaches. *American Journal of Dance Therapy*, *41*(2), 214-233.	Illinois	To integrate Dance Movement Therapy-informed approaches to help African American youth in Chicago's Roseland community understand, explore, and heal from Post Traumatic Slave Syndrome	Dance (active participatory)	N=4 (ages not given); 1 African American dance/movement therapist, 1 program developer, 1 arts and education director, 1 arts education manager	Report	Program increases in self-awareness and self-esteem, leadership roles and enrollment in one or more club or organization, sharing of knowledge with others within their community, and exploration of personal healing.		None received

Extracted Data for all Publications

Lamar, C.C. (2019). A Pathway Home: Connecting Museum Collections with Native Communities In *Arts* (pp. 154). Multidisciplinary Digital Publishing Institute. https://doi.org/10.3390/arts8040154	New Mexico	To facilitate community well-being for residents of the Tewa Pueblos of Pojoaque, Nambe, Ohkay, Owingeh, Santa Clara, San Ildefenso, and Tesuque via heritage preservation of pottery and storytelling	Community and Cultural (Participation in museums) (receptive participatory)	N=~650 (ages not given); further demographic data not given	Report	Collections in museums may contribute to the processes of cultivating revitalization and contribute to their society's well-being.	Positive	Margaret A. Cargill Philanthropies
O'Brien, E. (2016). Move2Love and vibrancy: Community dance/fitness. *Women & Therapy*, *39* (1-2), 171-185.	Pennsylvania	To describe the history of the Move 2 Love program, the elements of the protocol and the incorporation of positive psychology principles. And report participant's lived experiences in the program	Dance (active participatory)	N not given (ages 50-94); all female, further demographic data not given	Report	This program contributed to addiction avoidance in an at-risk population, lowered disease burden, and increased physical, social, and psychological well-being.	Positive	Not given
Pourat, N., Martinez, A. E., Haley, L. A., & Chen, X. (2018). Parks After Dark	California	Using cultural events in the parks to increase access to quality recreational	Mixed (community and cultural and music and visual arts and	N=~178,000 (ages <16 (24%), 17-21 (19%), >22 (66%); females (66%),	Report	Parks after Dark contributed to social cohesion and community	Positive	Not provided

Evaluation Brief. *Policy Brief (UCLA Center for Health Policy Research), 2018*(4), 1-12.		programming and innovative services; decrease community violence and increase perceptions of safety; increase physical activity and decrease chronic disease risk; improve social cohesion and community well-being; and reduce costs	craft) (active participatory)	Latinx (71%); income <$20k (52%)		well-being. The arts were only one part of the extensive activities the PAD program offered, and the results for well-being are not attributed to the arts alone.		
Sonke, J., Golden, T., Francois, S., Hand, J., Chandra, A., Clemmons, L., ... and Springs, S. (2019). Creating healthy communities through cross-sector collaboration [White paper]. *University of Florida Center for Arts in*	United States	Report with public health sector as a primary intended audience, it discusses the value of the arts and culture for advancing health and well-being in communities.	N/A	N not given (ages not given); further demographic data not given	Report	Presents art programs in the US that address collective trauma, racism, social exclusion/social isolation, mental health, chronic disease, and well-being.	N/A	Pabst Steinmetz Foundation and ArtPlace America

Extracted Data for all Publications

Medicine/ArtPlace America.								
