# Supplementary material for: Engaging the Arts for Wellbeing in the United States of America: A Scoping Review
Source: Front Psychol. 2022 Feb 9;12:791773. doi: 10.3389/fpsyg.2021.791773 (PMC8863598; doi:10.3389/fpsyg.2021.791773)
Supplement: Supplementary file 4 [file Presentation_1.PDF]

PRISMA Flow Diagram for the scoping review process adapted from the PRISMA statement by Moher and colleagues (2009).

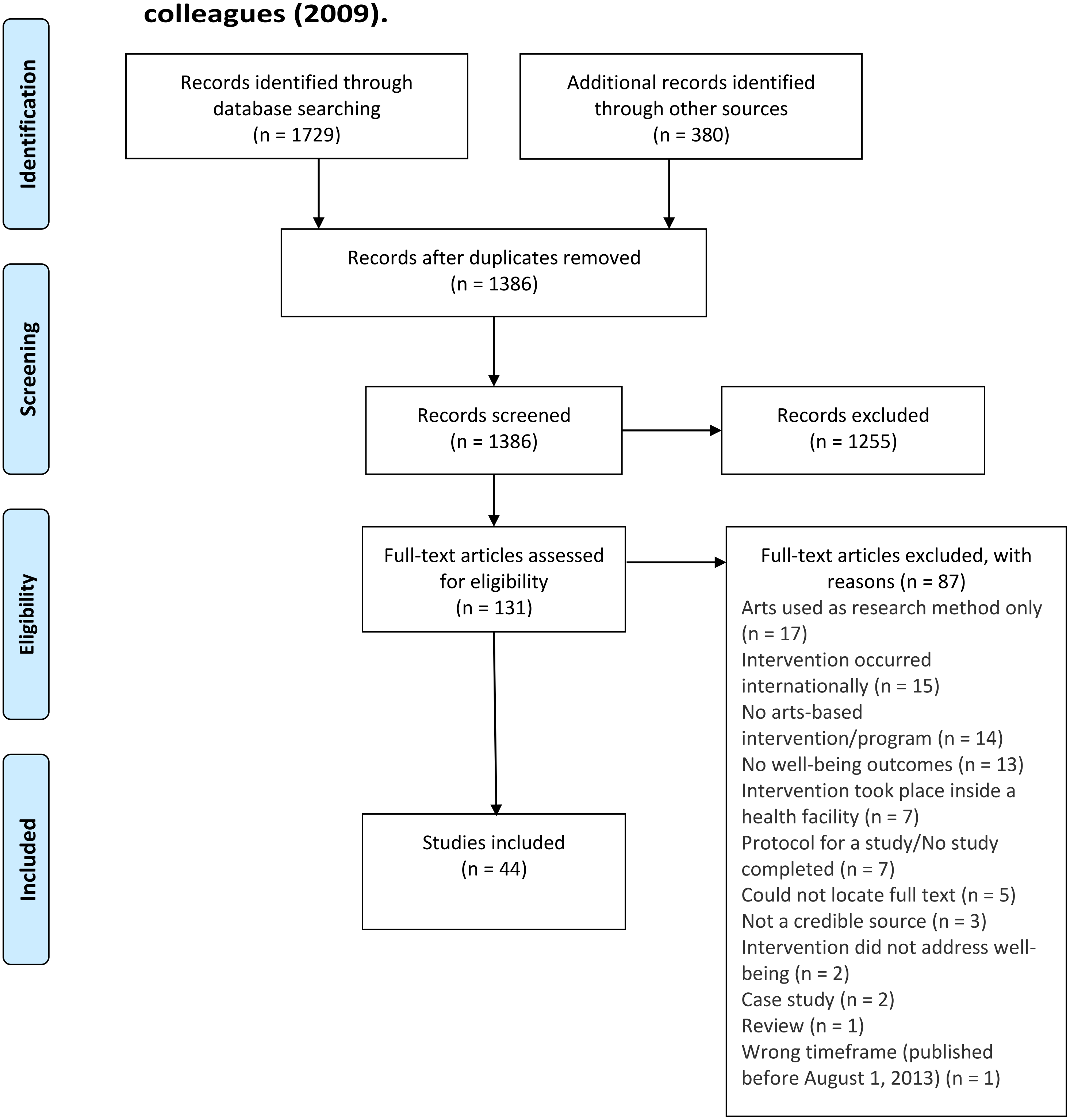

Adapted by: Aromataris E, Munn Z (Editors). JBI Manual for Evidence Synthesis. JBI, 2020. Available from https://synt hesismanual.jbi.global. https://doi.org/10.46658/JBIMES-20-01

From: Moher D, Liberati A, Tetzlaff J, Altman DG, The PRISMA Group (2009). *P*referred *R*eporting *I*tems for *S*ystematic Reviews and *M*eta-*A*nalyses: The PRISMA Statement. PLoS Med 6(7): e1000097. doi:10.1371/journal.pmed1000097

For more information, visit www.prisma-statement.org.
